# The clinical application of electrical impedance technology in the detection of malignant neoplasms: a systematic review

**DOI:** 10.1186/s12967-020-02395-9

**Published:** 2020-06-08

**Authors:** Angela A. Pathiraja, Ruwan A. Weerakkody, Alexander C. von Roon, Paul Ziprin, Richard Bayford

**Affiliations:** 1 Department of Surgery and Cancer, Imperial College London, London, UK; 2 St Mary’s Hospital, 10th Floor QEQM Building, Paddington, London, W2 1NY UK; 3 Department of Natural Sciences, Middlesex University, London, UK; 4 School of Science and Technology, Middlesex University, The Burroughs, Hendon, London, NW4 4BT UK

**Keywords:** Malignant, Neoplasms, Cancer, Real-time, Detection, Diagnosis, Electrical impedance, Spectroscopy

## Abstract

**Background:**

Electrical impedance technology has been well established for the last 20 years. Recently research has begun to emerge into its potential uses in the detection and diagnosis of pre-malignant and malignant conditions. The aim of this study was to systematically review the clinical application of electrical impedance technology in the detection of malignant neoplasms.

**Methods:**

A search of Embase Classic, Embase and Medline databases was conducted from 1980 to 22/02/2018 to identify studies reporting on the use of bioimpedance technology in the detection of pre-malignant and malignant conditions. The ability to distinguish between tissue types was defined as the primary endpoint, and other points of interest were also reported.

**Results:**

731 articles were identified, of which 51 reported sufficient data for analysis. These studies covered 16 different cancer subtypes in a total of 7035 patients. As the studies took various formats, a qualitative analysis of each cancer subtype’s data was undertaken. All the studies were able to show differences in electrical impedance and/or related metrics between malignant and normal tissue.

**Conclusions:**

Electrical impedance technology provides a novel method for the detection of malignant tissue, with large studies of cervical, prostate, skin and breast cancers showing encouraging results. Whilst these studies provide promising insights into the potential of this technology as an adjunct in screening, diagnosis and intra-operative margin assessment, customised development as well as multi-centre clinical trials need to be conducted before it can be reliably employed in the clinical detection of malignant tissue.

## Background

Over the last 30 years, oncological treatment strategies have been efficiently developed, researched and streamlined to enable rapid diagnosis and effective management of malignant disease. More recently there has been particular interest into biotechnology that can further improve the diagnosis and surgical management of malignancies, especially in non-/minimally-invasive ways; examples include breath biomarkers of cancer, minimally-invasive surgical strategies such as robotic and transanal surgery, as well as surgical margin technologies [1–5]. Consequently, an increasing body of research into the clinical application of electrical impedance techniques has been emerging.

Electrical impedance is a well-established physical concept in which an object’s impedance (a surrogate calculated measure of electrical conductivity) to an applied alternating current over increasing frequencies can be measured, in order to assess tissue composition [6]. Whilst it has been used extensively in the engineering domain, over the last 30 years there has been increasing interest into its use and application in the medical world. Resultingly this technology is now utilized in everyday clinical practice in intensive care and nutritional medicine, in the measurement of fluid volumes as well as overall fluid status and body composition [6, 7].

**Fig. 1:**
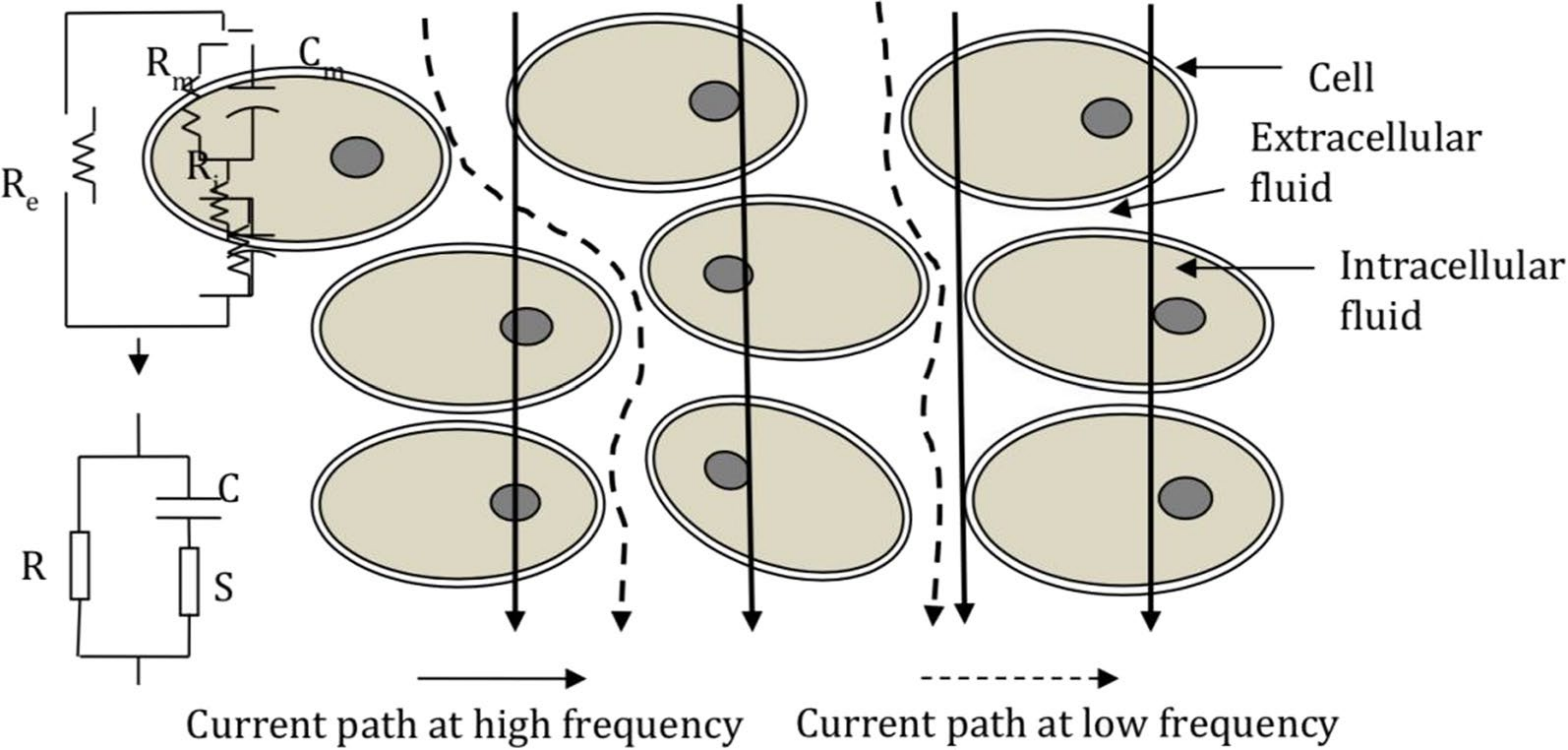
Diagram showing how conduction alters with electrical and structural changes

It is known that tissue conductivity and impedance changes as cell structure, fluid status and electrical current alters, as seen in Fig. 1 [8]. Due to this unique property, there has been a lot of inquiry into how electrical impedance changes as tissue becomes pathological, specifically malignant. It has been hypothesized that as a malignant pathological process causes transformation of the cellular structure, the tissue’s electrical conductivity and therefore electrical impedance would also change [9]. Over the recent years there has been renewed interest and research looking at electrical impedance changes in various cancer subtypes. Although there is some understanding of the pathological processes underlying these changes, there has yet to be a general consensus on how exactly tissue electrical impedance changes with the development of a malignant process [10].

Therefore, the objective of this systematic review was to compare the results from studies looking at electrical impedance changes in various cancer subtypes, in order to assess this technology’s potential application in the detection of malignant neoplasms.

## Methods

A qualitative systematic review was undertaken using the Preferred Reporting Items for Systematic Reviews and Meta-analysis (PRISMA) guidance. Embase Classic, Embase, Psychinfo and Medline search engines were used to perform a systematic literature review between 1980 and 22/02/2018. The following search terms were used with the Boolean operators OR: bioimpedance, electrical impedance spectroscopy, electrical impedance tomography. These results were then searched for “cancer or oncology” and duplication bias studies were removed. The abstracts of the remaining studies were then independently analysed (by AAP and RAW) to identify studies eligible for inclusion. Those studies that were written in the English language were evaluated against the following inclusion criteria:

- Studies of human adult tissue
- Studies where either in vivo tissue or ex vivo freshly resected tissue was tested
- Comparative studies evaluating electrical impedance of cancer against normal tissue
- Comparative studies where histopathological tissue diagnosis of the different tissue samples tested was used as the reference point.

Conference abstracts as well as general abstracts which had not progressed to full published articles were excluded. The references of those articles that met the inclusion criteria were then cross-referenced to ensure that no other studies were missed. The data from all the eligible studies were then computerised and collated. As there was considerable heterogeneity between the various included studies, the articles that were relevant to the review were classified according to their cancer subtypes, and a qualitative analysis of the data set was made, with the primary endpoint of the studies being defined as the ability to distinguish between normal and pre-malignant and/or malignant tissue. Other clinically relevant results were also identified and reported.

For each study AUROC (area under the receiver operating characteristic curve), sensitivity and specificity data were collected where available. Where AUROC data was not available, a standard composite measure of sensitivity and specificity was used: Youden index, calculated as (sensitivity + specificity) minus 1 [11]. This is an established surrogate measure, representing the vertical distance between a putative AUROC curve and the equal line [11]. In order to compare the studies, the discriminative strengths were then qualitatively categorised as: “Good”, AUROC > 0.7 or sensitivity and specificity both > 0.75 or Youden index > 0.5; “Moderate”, AUROC > 0.6 or Sensitivity > 0.7 and Youden index > 0.25; “Insufficient”, not meeting the aforementioned criteria or reporting insufficient data. For each cancer type, a qualitative categorisation of the body of evidence was made, based on the discriminative strength of each study as well as the size and number of studies.

## Results

**Fig. 2:**
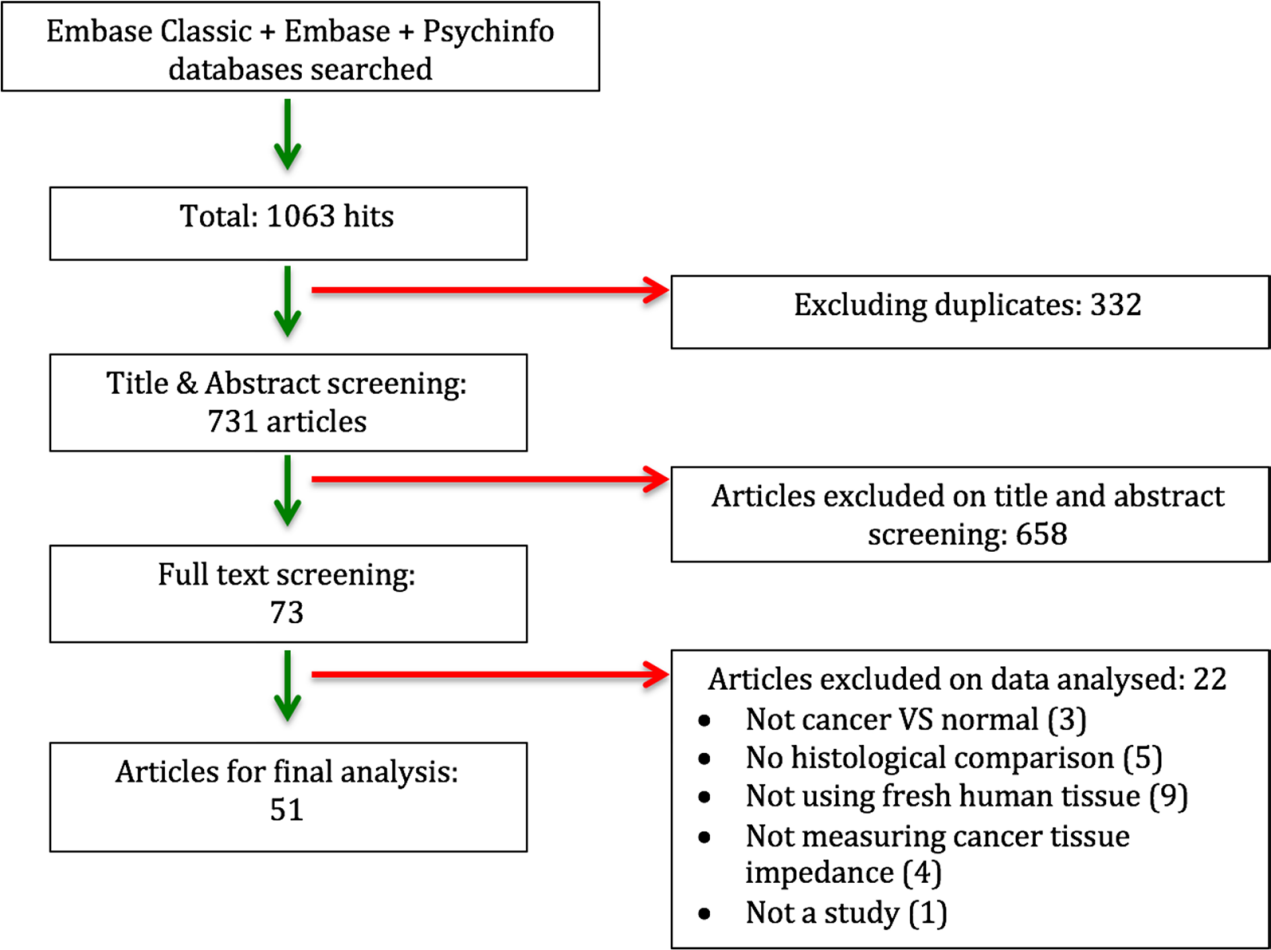
PRISMA flow chart of study selection

From 731 abstracts identified, 73 articles met the inclusion criteria, of which 51 had sufficient data that could be analysed. Figure 2 shows the PRISMA flowchart employed for this review.

51 articles covering the study of electrical impedance spectroscopy (EIS) on 16 different malignant tissue types were analysed. These covered a total of 7035 patients’ specimens analysed by EIS in 16 different cancer types (compared with corresponding normal tissue). 29 studies were published after 2010, and 10 of these were published in 2015 or after. All 4 cutaneous melanoma studies, 1 breast study and 1 hepatic study were conducted as multicentre studies; all other studies (45)  were conducted at single sites. All 4 cervical studies, both tongue studies and all except 1 of the 12 prostate studies were conducted at the same institution. 23 of the studies were conducted in the USA and 15 in Europe.

Table 1 describes the outcomes of in vivo studies reporting on pre-malignant and malignant tissue states. Table 2 shows the results of all the other studies investigating impedance in malignant tissue types only. Table 3 provides summative results of all 51 studies by cancer type, and a Additional file 1: Table S1 details key secondary outcomes for all the studies analysed in the review.

**Table 1:** Results of in vivo studies of pre-malignant and malignant tissue

Author	Country	Histology of pre-/malignant tissue	No. of pre-/malignant samples	No. of normal (control) samples	AUROC	Sensitivity (%)	Specificity (%)	Discriminative strength^a^
Tidy 2013	UK	High-grade CIN	87	109	0.827	86	56	Good
Balasubramani 2009	UK	High-grade CIN	85	681	0.800	79	73	Good
Brown 2000	UK	High-grade CIN	126	370	0.951	92	92	Good
Abdul 2006	UK	High-grade CIN	178	680	0.652	74	53	Moderate
Malvehy 2014	Multi-centre	Melanoma, NMSC	320	320	NR	97	34	Moderate
Mohr 2013	Multi-centre	Melanoma, NMSC	126	126	NR	98	NR	Insufficient
Aberg 2011	Multi-centre	Melanoma	59	59	0.850	95	49	Good
Rocha 2017	Multi-centre	Melanoma	6	154	NR	100	70	Good
Murdoch 2014	UK	Oral SCC	10	61	0.776	65	92	Good
Sarode 2015	India	Oral SCC	50	50	NR	NR	NR	Insufficient

AUROC: area under receiver operating characteristic curve for discrimination between neoplasia and normal tissue; CIN: cervical intraepithelial neoplasia; NMSC: non-melanomatous skin cancer; Melanoma refers to cutaneous melanoma in all cases. SCC: squamous cell carcinoma; NR: not reported

^a^Discriminative strength is a summary of the reported quantitative indices for discrimination between neoplastic and normal tissue in each study, not taking into account the number of samples. This was rated as follows: “Good”, AUROC > 0.7 or sensitivity and specificity both > 0.75 or Youden index > 0.5; “Moderate”, AUROC > 0.6 or Sensitivity > 0.7 and Youden index > 0.25; “Insufficient”, not meeting the above criteria or insufficient data. Youden index is calculated as the (sensitivity + specificity) minus 1 [11]

**Table 2:** Results of other studies comparing malignant neoplasm versus normal tissue (grouped by cancer type)

Author	Country of origin	Site	Histology of malignant tissue tested	Cancer samples	Benign samples	Specimen state	Discriminative strength^a^
Halter 2015	USA	Breast	IDC	10	9	In-vivo	Good
Gregory 2012	USA	Breast	IDC or ILC ± DCIS or LCIS	232	141	Ex-vivo	Insufficient
Kim 2007	USA	Breast	IDC	1	1	In-vivo	Insufficient
Cherepenin 2001	Russia	Breast	–	21	21	In-vivo	Insufficient
da Silva 2000	Portugal	Breast	–	21	42	Ex-vivo	Insufficient
Osterman 2000	USA	Breast	ILC or IDC	3	12	In-vivo	Insufficient
Chauveau 1999	France	Breast	IDC	2	1	Ex-vivo	Insufficient
Jossinet 1998	France	Breast	–	23	64	Ex-vivo	Insufficient
Stojadinovic 2005	Multicentre	Breast	(Various)	29	1074	Ex-vivo	Good
Kao 2008	USA	Breast	IDC and DCIS	3	1	In-vivo	Moderate^b^
Halter 2009	USA	Breast	IDC + DCIS	11	4	Both	Insufficient
Du 2017	China	Breast	(Various)	581	395	Ex-vivo	Good
Mahara 2015	USA	Prostate	–	3	3	Ex-vivo	Insufficient
Mishra 2013	USA	Prostate	–	21	367	Ex-vivo	Good
Wan 2013	USA	Prostate	–	45	45	In-vivo	Insufficient
Mishra 2012	USA	Prostate	–	36	288	Ex-vivo	Insufficient
de Abreu 2011	Brazil	Prostate	–	23	27	In-vivo	Insufficient
Halter 2011	USA	Prostate	–	71	465	Ex-vivo	Good
Halter 2008	USA	Prostate	–	29	151	Ex-vivo	Insufficient
Halter 2007	USA	Prostate	Adenocarcinoma	o	5	Ex-vivo	Insufficient
Khan 2016	USA	Prostate	–	23	53	Ex-vivo	Insufficient
Murphy 2017	USA	Prostate	–	12	105	Ex-vivo	Good
Lee 1999	USA	Prostate	–	6	6	Ex-vivo	Insufficient
Halter 2008	USA	Prostate	–	17	345	Ex-vivo	Insufficient
Keshtkar 2006	Iran	Bladder	–	24	73	In-vivo	Insufficient
Wilkinson 2002	UK	Bladder	–	35	35	Ex-vivo	Insufficient
Keshtkar 2012	Iran	Bladder	–	30	100	Ex-vivo	Good
Prakash 2014	USA	Hepatic	Metastasis-colorectal primary	41	91	Ex-vivo	Insufficient
Laufer 2010	Israel & USA	Hepatic	–	32	26	Ex-vivo	Insufficient
Gao 2014	China	Lung	(Various)	91	91	Ex-vivo	Insufficient
Cherepenin 2001	Russia	Lung	–	22	7	In-vivo	Insufficient
Sun 2010	Taiwan	Tongue	SCC	12	12	In-vivo	Insufficient^c^
Ching 2010	Taiwan	Tongue	SCC	5	5	In-vivo	Insufficient
Dua 2004	USA	Skin -BCC	BCC	18	16	In-vivo	Insufficient
Kuzmina 2005	Sweden	Skin -BCC	BCC	35	35	In-vivo	Insufficient
Keshtkar 2012	Iran	Gastric	Adenocarcinoma	19	22	In-vivo	Insufficient
Yun 2016	South Korea	Renal	RCC	10	10	Ex-vivo	Insufficient
Zheng 2013	USA	Thyroid	Papillary & follicular	27	133	In-vivo	Good
Pathiraja 2017	UK	Colorectal	Adenocarcinoma	22	22	Ex-vivo	Good
Habibi 2011	USA	Skin-scc	SCC	1	14	In-vivo	Insufficient
Knabe 2013	Germany	Oesophagus	Adenocarcinoma & SCC	30	19	In-vivo	Insufficient^c^

IDC: invasive ductal carcinoma; ILC: invasive lobular carcinoma; DCIS: ductal carcinoma in situ; LCIS: lobular carcinoma in situ; SCC: squamous cell carcinoma; BCC: basal cell carcinoma; (Various): more than 3 histological cancer subtypes included; ‘–’, not stated

^a^Discriminative strength is a summary of the reported quantitative indices for discrimination between neoplastic and normal tissue in each study, not taking into account the number of samples. This was rated as follows: “Good”, AUROC > 0.7 or sensitivity and specificity both > 0.75 or Youden index > 0.5; “Moderate”, AUROC > 0.6 or Sensitivity > 0.7 + Youden index > 0.25; “Insufficient”, not meeting the above criteria or insufficient data. Youden index is calculated as (sensitivity + specificity) minus 1 [11]

^b^This study reported moderate discriminative ability in the < 40 year group

^c^These two studies reported statistically significant differences between malignant and normal tissue on EIS, but insufficient data for calculating the overall discriminative strength

**Table 3:** Summative results of the 51 studies by cancer type

Site of malignancy	Histopathological type	No. of studies	Total no. of patients	Specimen state	Summative evidence
Cervical	Cervical SCC	4	638	In-vivo	Strong^a^
Prostate	–	12	297	Both	Strong
Skin	Skin melanoma & NMSC	7	3009	In-vivo	Moderate
Breast	Various carcinomas	12	2460	Both	Moderate
Oral mucosa	SCC	2	198	In-vivo	Moderate
Bladder	–	3	123	Both	Moderate
Thyroid	Various carcinomas	1	27	In-vivo	Moderate
Colorectal	Adenocarcinoma	1	22	Ex-vivo	Moderate
Tongue	SCC	2	29	In-vivo	Weak
Lung	Various	2	138	Both	Weak
Hepatic	Metastases	2	16	Ex-vivo	Weak
Oesophagus	SCC & adenocarcinoma	1	23	In-vivo	Weak
Gastric	Adenocarcinoma	1	45	In-vivo	Weak
Renal	Renal cell carcinoma	1	10	Ex-vivo	Weak

SCC: squamous cell carcinoma; NMSC: non-melanomatous skin cancer; BCC: basal cell carcinoma; ‘–’, not stated

Summative evidence strength classified as “Strong”: 2 or more large studies with good discriminative ability

“Moderate”: good discriminative indices described but in small numbers for any specific histological type

“Weak”: insufficient statistical data to demonstrate significant discriminative ability currently

### Cancer subtypes

#### Breast cancer

Twelve articles described studies into breast cancer on a total of 2460 patients. Six of the 12 studies looked at in vivo tissue by measuring the electrical impedance and electrical properties of the overlying skin or lesion itself intra-operatively [12–17], whilst the other six studies directly measured impedance of fresh ex vivo specimens [18–23]. Three studies looked at intra-ductal carcinoma (IDC) alone [12, 13, 20], whilst six studies looked at various different carcinoma subtypes [14–17, 21, 22], and the remaining three studies did not report the exact histopathology of the cancer specimens tested [14, 19, 21]. All the studies noted statistically significant changes in various electrical impedance metrics of malignant tissue, and 2 of the studies went on to use the metrics to form a classification by which they were able to accurately discriminate tissue based on the classification results [19, 23]. One study was able to integrate the various parameters (R_o_/R_∝_ and f_c_) to create a new parameter that was able to distinguish malignant tissue with an AUROC of 0.93 [24]. Four of the breast studies used EIS technology either integrated or in combination with mammographic technology in order to produce images as well as quantitative measurements of the tissue [13, 15–17]. One study was able to show that impedance was affected by age and hormonal state of the patient and was most sensitive at identifying cancers in patients under 40 without any palpable lesions [22]. In addition to the quantitative results reported in Table 2, most of the studies also concluded that as the technology was found to be painless, non-invasive and radiation-free, it had the potential to be developed into a useful pre-screening tool.

#### Prostate cancer

Twelve articles looked at changes in electrical impedance related to prostate cancer on a total of 297 patients, with only two of the studies examining in vivo tissue [24, 25]. Of the two in vivo studies, one applied an ultrasound probe that had integrated electrical impedance technology, so that measures could be taken from the probe prior to surgical excision of the tissue [24]. The second in vivo study measured whole body electrical impedance and chronoamperometry in patients with and without cancer, in order to determine impedance differences between the 2 groups, as well as improved detection rates when combining electrical impedance measures with PSA levels [25]. Additionally, the two in vivo studies used EIS devices that were able to produce images that could identify malignant lesions. All 12 studies showed statistically significant differences in impedance metrics between normal and malignant prostatic tissue, and 1 study was also able to discriminate between low-grade and high-grade malignant tissue as well [26]. One study showed that different histopathologies produced different EIS spectra, as well as statistically significant differences in tissue resistivity between malignant and benign prostatic hypertrophic tissue [27]. 11 of the studies were conducted at the same institution, employing similar tools and methodology, in which the electrical impedance measurement was integrated into the prostate biopsy device [24, 26–34]. Three of the studies report on optimal frequencies noted for discrimination of tissue; however, all the frequencies reported were found to be different. All 11 studies from the same institution found that the electrical impedance was significantly higher in prostate cancer tissue compared to normal prostatic tissue. The results from the prostate studies suggest that such technology could be developed to provide a real-time screening tool and even an adjunct to PSA testing in prostate cancer screening programmes.

#### Cervical cancer

Four large in vivo studies looked at electrical impedance changes found at colposcopy of suspicious cervical lesions on a total of 638 patients. All these studies were conducted at the same centre, using the same tools and methodology [10, 35–37]. All four studies showed that electrical impedance measurements were able to reliably discriminate high-grade cervical intra-epithelial neoplasm (CIN) from normal cervical tissue, with AUROC ranging from 0.652–0.951. The accuracy of detection of pre-malignant lesions was relatively unaffected by the application of acetic acid to the tissue (which is routinely done during colposcopic assessment of suspicious cervical lesions) and was generally found to be very sensitive but less specific in lesion identification [36]. All 4 studies suggest that as the CIN grade increases, the impedance and resistivity decrease whilst conductivity increases. The studies also found that combining EIS measurement with colposcopy increases the accuracy of the detection of high-grade CIN [35]. Further statistical analysis of the results from all 4 studies showed the following weighted means: AUROC of 0.8, sensitivity of 0.81, and specificity of 0.71.

#### Skin cancer

4 large-scale multicentre trials involving 2933 patients looked at electrical impedance changes found in cutaneous melanomas. All the trials were conducted in vivo, using a similar methodology [38–41]. All the studies showed the electrical impedance technology was able to distinguish both melanomatous and non-melanomatous skin tumours with very high sensitivities > 95%. They also noted that the sensitivity of the technique increased further as the Breslow thickness of the malignant tissue increased. All the studies were also able to identify statistically significant differences between normal tissue, non-malignant atypical lesions and non-melanomatous skin cancers. Additionally, 1 study was able to combine dermoscopy results with a higher EIS cut-off level to identify malignant lesions with 100% sensitivity [38].

#### Oral cancer

Four studies looked at two types of oral lesions: two studies tested in vivo oral SCC lesions in 198 patients [42, 43], and two tested in vivo SCCs occurring on the tongue in 29 patients, both of which were conducted at the same institution [44, 45]. All four studies reported that oral and tongue SCCs had much lower impedance measures than normal oral and tongue epithelium respectively and could therefore be identified. Both studies of tongue cancer as well as one of the oral SCC studies showed statistically significant differences in impedance were most pronounced between 20 Hz and 50 kHz [43–45]. The two studies of tongue cancers also showed differences in other impedance metrics between normal and malignant tongue lesions.

#### Bladder cancer

3 studies looked at electrical impedance changes in bladder malignancy of 123 patients [46–48], of which one study was an in vivo investigation [46]. All three studies found that malignant urothelium had higher electrical impedance than normal urothelium, and one study was able to record an AUROC of 0.887, showing good separation between normal and malignant tissue [48]. Additionally, one of the studies noted important effects of oedema and inflammation on the impedance results [47]. All three studies suggest that following further development of the customised technology, electrical impedance could play a role in assisting in cystoscopic assessment and screening of suspicious urothelial lesions.

#### Hepatic cancer

Two studies looked at the electrical impedance of ex vivo hepatic malignancies in 16 patients; one of the studies focussed on primary malignancy [49] whilst the other study looked at hepatic metastasis from a colonic primary cancer [50]. Both studies showed that the malignant tissue exhibited a much higher conductivity than the normal tissue, and consequently a much lower impedance than normal tissue. One of the studies highlighted the importance of malignant tissue’s higher conductivity in relation to radiofrequency (RF) ablation of hepatic tissue: lowering RF ablation frequencies may enable better targeting of the malignant tissue alone, without surrounding normal tissue conducting and consequently being damaged by RF ablation [49].

#### Lung cancer

Two articles looked at lung malignancies of various pathological subtype in 138 patients. Both studies were able to show that various measured electrical impedance metrics were able to discriminate lung cancer from normal healthy lung tissue [51, 52]. In particular lung cancer showed a statistically significant increase in conductivity compared to normal tissue. One study goes further by suggesting that its method of static electric impedance tomography could potentially be developed into a screening tool for lung cancer [52].

#### Basal cell carcinoma

Two articles looked at electrical impedance changes in in vivo basal cell carcinomatous tissue on a total of 61 patients. In both studies various EIS parameters could accurately distinguish BCC from normal tissue with statistical significance, as well as consistently lower impedance measurements in BCCs compared to normal tissue [53, 54]. One study compared measuring the lesion’s impedance along with its surrounding skin, against the lesion alone, and was able to show 100% vs. 85% sensitivity respectively [53]. The second study additionally looked at laser Doppler and transepidermal water loss measurements as well as impedance measures and found similar trends between all 3 sets of measurements, but was unable to discriminate between the nodular- and superficial subtype of the lesion [54].

#### Individual studies of other cancers

Six other independent studies looked at the effects of the malignant process on the electrical conductivity of various tissue types, four of which were on in vivo specimens. The 6 individual tissue types included thyroid malignancies, squamous cell carcinomas of the skin, oesophageal adenocarcinoma and squamous cell carcinoma, gastric adenocarcinoma, colorectal adenocarcinoma and renal cell carcinoma [55–60]. Whilst these six individual studies were generally of a smaller malignant tissue sample size, all six studies were able to show that various bioimpedance parameters could be used to distinguish malignant from normal tissue. The studies of gastric adenocarcinoma, renal cell carcinomas, various thyroid malignancies and squamous cell skin cancer all showed malignant tissue to have lower impedance and/or resistivity/permittivity than normal [55–58], whilst the colorectal and oesophageal studies showed higher impedance with malignant lesions compared to normal [58–60]. Additionally, the colorectal study showed that in two previously malignant lesions that had shown complete response to chemoradiotherapy, the impedance measurements appeared to be similar to that of normal colorectal tissue [58].

Two cancer groups’ evidence—cervical and prostate cancer—were categorised as “strong”, having two or more large studies with strong discriminative variables, good reference standards and homogenous methodologies between studies. Six cancer types were deemed to have “moderate” evidence; these included skin, breast, oral, bladder, thyroid and colorectal cancer. Although there were multiple studies on various breast, skin and oral cancers, only two studies on breast cancer, two studies on melanoma, and one study on oral cancer showed good discriminative ability [12, 23, 40–42]. The studies on bladder, thyroid and colorectal cancer showed promising early results in terms of discriminative indices, but had small sample sizes [46–48, 57, 58]. The 6 remaining cancer types—tongue, lung, hepatic metastasis, oesophageal, gastric and renal—were categorised as having “weak” evidence as the studies reported insufficient data: either not enough quantitative data, or other metrics that could not be quantified.

## Discussion

The aim of this study was to evaluate the clinical application of electrical impedance technology in the detection of malignant neoplasms. A systematic review of the available published data over the last 36 years identified 51 articles that studied the effect of malignancy on tissue electrical impedance and its associated metrics, which have been reported in this paper.

All the studies reported differences in electrical impedance and/or related metrics between the normal tissue and corresponding malignant tissue. However, the actual change in the impedance itself appeared to vary depending on the histopathological cancer that was measured. As the electrical impedance spectroscopy and tomography technology generates various metrics including conductivity, resistivity, and impedance/admittivity, as well as tomographic spatial images, various changes in these different metrics were noted. It was noted that the majority of the squamous cell carcinomatous tissue seemed to result in significantly lower impedance spectra, in contrast with the adenocarcinomatous tissue studies, which gave a more variable spread of impedance changes. This finding was consistently seen throughout all the studies of cervical, skin and oral squamous cell carcinomas (SCC), where the SCC tissue gave significantly lower SCC readings than its corresponding normal tissue. Conversely, adenocarcinomatous tissue types seen in the prostate, breast, oesophageal and colorectal studies showed more variable results. Whilst the breast and oesophageal studies showed lower impedance levels in the adenocarcinomatous subtypes of tissue examined, the prostate and colorectal studies showed significantly higher electrical impedance levels in the malignant tissue. Additionally, the colorectal study showed that in a small subset of specimens that had undergone complete response to chemoradiotherapy, the resulting impedance appeared to have returned to a lower impedance, similar to that of normal colorectal tissue. It is unclear as to why these differences occur. However, as none of the studies look at the individual histopathological states on a microscopic level, correlation between the cellular changes and impedance changes could not be established at this stage. Additionally, as the studies on the adenocarcinomatous tissues were of a much smaller magnitude than the much larger-scale cervical and cutaneous melanoma studies, it is difficult to make firm conclusions based on these results alone.

In the studies of both cervical tissue and cutaneous skin lesions, the electrical impedance technology was so sensitive that it was able to discriminate between normal and pre-malignant tissue as well as normal and malignant tissue, with AUROC analysis of over 95% and 85% respectively. These findings are particularly significant as the cervical and cutaneous data sets represent a very large sample size of 638 and 2933 patients respectively. Additionally, the cutaneous lesion data sets originate from four separate multi-centre trials, which all showed similar trends in very high sensitivities, with comparatively lower specificities. As these large-scale studies have shown such significant results, they strongly suggest the potential diagnostic accuracy of real-time diagnosis of cervical CIN and malignant skin lesions, using electrical impedance technology.

Another interesting point to note was that several different studies exploited the EIS technology in order to tailor the parameters measured and/or the ergonomics of the tool for the specific cancer in question. One study in the breast cancer subgroup had specifically designed their EIS algorithms to be most accurate for breast tissue of women under 40, whilst another study in the prostate subgroup integrated the EIS technology onto the end of an endoanal ultrasound probe in order to access and measure the field of interest. Additionally, several research groups in breast and prostate used the technology to produce real-time images (“maps”) that correlated directly with the anatomy being measured. These studies show the great potential of this technology, not only in the way that the EIS metrics can be specifically tailored and displayed, but also in the breadth of application of this compact, mobile and cheap electrode technology in various clinically appropriate forms.

One major limitation that should be noted in this review is that several of the studies (25/51) included in this systematic review used ex vivo specimens; only the cervical, cutaneous and oral lesion studies exclusively looked at electrical impedance on in pre-/malignant and normal tissue. One of the breast studies included a comparison between in vivo and ex vivo measurements of specimens and showed that various EIS metrics (conductivity and permittivity) clearly decrease as measurements are taken ex vivo. It is known that as soon as tissue is resected and loses its blood supply, the fluid status of the tissue changes, which in turn would affect the electrical conductivity and impedance properties of the tissue. However, it is understandable that in these initial proof-of-concept studies where novel technology and techniques are being used for the first time, ex vivo studies precede more realistic in vivo studies. Therefore, further research examining the electrical impedance of these tissue types in vivo would be required before an assessment of the effectiveness of this technology could be made.

Another limitation to consider is that many of the studies included have a small sample size, and have each reported on different outcomes, which therefore could not be statistically analysed as a whole. This heterogeneity is increased by the studies having multiple variables, such as frequency ranges applied by the studies’ tools, the specific impedance tool used as well as unreported ischaemic times. For the cancer types that have many studies reporting findings, the studies have often been conducted at the same institution using the same methodology but have not reported quantitative statistics that could be pooled for analysis. Consequently, more meaningful statistical analysis of the results could not be reported at this early stage. Nevertheless, qualitative analysis of the results was still possible, from which significant conclusions and further work can be planned.

## Conclusions

This is the first systematic review into the application of electrical impedance technology on normal, pre-malignant and malignant tissue types. Although the studies are mostly heterogeneous in their methodologies and findings, they do provide evidence of the technical feasibility of electrical impedance technology for differentiating between normal, pre-malignant and malignant lesions in a range of tissue types. This has the potential to be exploited as a diagnostic adjunct, for example as demonstrated by the use of EIS in colposcopy for the assessment of cervical abnormalities; in the latter case though research into long-term outcomes is recommended and remains to be seen [61]. Additionally, in the context of endoscopic, robotic and artificial intelligence technology, electrical impedance has the potential to be integrated into these technologies to further augment their capabilities [62, 63].

Further histopathological analysis needs to occur alongside analysis of the impedance data, in order to fully understand why these changes are occurring. Through better understanding of the underlying malignant structural differences causing the changes in electrical impedance, this technology could be specifically enhanced both technically and ergonomically, in order to examine these changes more accurately. In order for this technology to be more rigorously assessed in the future, large-scale multicentre in vivo studies will be needed, in order to calculate the optimal tools and metrics that give the most accurate rates of cancer detection. Additionally, the effects that these technologies’ results have on clinical decision-makers’ behaviours as well as the patients’ long-term outcomes will need to be investigated in future studies. If this novel technology can be further developed and found to improve detection rates and surgical outcomes, these highly sensitive and non-invasive real-time diagnostic tools could be implemented throughout various different clinical settings (ranging from bedside diagnosis to intra-operative margin assessment), and thereby provide point-of-care testing, real-time diagnosis and effective surgical management of the patient’s cancer in an efficient and cost-effective manner.

## Supplementary information

**Additional file 1. Table S1.** Detailed secondary outcomes of all studies.

## Data Availability

All data and materials related to this article were explicitly referenced in the paper and can be obtained from the corresponding author.
